# Noninvasive Ventilatory Correction as an Adjunct to an Experimental Systemic Reperfusion Therapy in Acute Ischemic Stroke

**DOI:** 10.4061/2010/108253

**Published:** 2010-10-31

**Authors:** Kristian Barlinn, Clotilde Balucani, Paola Palazzo, Limin Zhao, April Sisson, Andrei V. Alexandrov

**Affiliations:** ^1^Comprehensive Stroke Center, Department of Neurology, University of Alabama at Birmingham, RWUH M226, 619 19th Street South, Birmingham, AL 35249-3280, USA; ^2^Dresden University Stroke Center, University of Technology Dresden, 01307 Dresden, Germany

## Abstract

*Background*. Obstructive sleep apnea (OSA) is a common condition in patients with acute ischemic stroke and associated with early clinical deterioration and poor functional outcome. However, noninvasive ventilatory correction is hardly considered as a complementary treatment option during the treatment phase of acute ischemic stroke. *Summary of Case*. A 55-year-old woman with an acute middle cerebral artery (MCA) occlusion received intravenous tissue plasminogen activator (tPA) and enrolled into a thrombolytic research study. During tPA infusion, she became drowsy, developed apnea episodes, desaturated and neurologically deteriorated without recanalization, re-occlusion or intracerebral hemorrhage. Urgent noninvasive ventilatory correction with biphasic positive airway pressure (BiPAP) reversed neurological fluctuation. Her MCA completely recanalized 24 hours later. *Conclusions*. Noninvasive ventilatory correction should be considered more aggressively as a complementary treatment option in selected acute stroke patients. Early initiation of BiPAP can stabilize cerebral hemodynamics and may unmask the true potential of other therapies.

## 1. Introduction

Obstructive sleep apnea (OSA) is the most common form of sleep disordered breathing in patients with acute ischemic stroke and associated with early neurological deterioration, increased mortality rates, and poor functional outcomes [1, 2]. Given that the rate of neurological deterioration is the highest during the acute stroke setting [3, 4], urgent treatment with noninvasive ventilatory correction may provide a novel therapeutic target to improve outcomes [5].

## 2. Case Report

**Figure 1:**
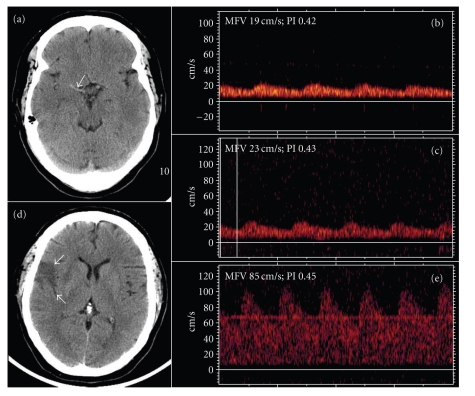
(a) Baseline noncontrast CT: a long thromboembolus within the right MCA trunk (arrow). (b) Baseline TCD: flattened systolic flow acceleration (TIBI 2) at a depth of 48 mm, indicating right proximal MCA occlusion. (c) One-hour TCD: improved flow velocities/waveforms (TIBI 2) at a depth of 58 mm suggestive of slow and partial recanalization. (d) Followup CT reveals a small cortical infarction (arrows). (e) 24-hour TCD: normal flow velocities and waveforms (TIBI 5) at a depth of 46 mm.

A 55-year-old female (weight 233 pounds, height 5 feet 7.75 inches, and body mass index 35.7) with a history of untreated OSA and arterial hypertension woke up with fluctuating right-hemispheric stroke symptoms. On admission, she had mild left-sided hemiparesis and facial palsy with dysarthria (National Institutes of Health Stroke Scale (NIHSS) Score 6). Cerebral computed tomography (CT) revealed early ischemic changes involving less than 1/3 of the right anterior middle cerebral artery (MCA) distribution and a hyperdense MCA sign (Figure 1). Transcranial Doppler (TCD) demonstrated a blunted flow of the right proximal/middle MCA (Thrombolysis in Brain Ischemia (TIBI) score 2, Figure 1) indicating an MCA occlusion. We initiated intravenous thrombolysis with tissue plasminogen activator (tPA) 200 minutes after symptom onset. After initiation of intravenous tPA infusion, the patient was enrolled in a thrombolytic research study (involving a direct thrombin inhibitor, permission of the sponsor was obtained to release this case report). 

At the end of tPA infusion, the patient developed excessive sleepiness with repetitive episodes (each lasting approximately 30 seconds) of irregular breathing and desaturation (with oxygen saturation levels below 90% under 2 to 4 liters supplemental oxygen delivered by a nasal cannula), and her neurological symptoms worsened rapidly (in less than 1 minute) to complete left-sided hemiplegia (NIHSS score 24). Her blood pressure (BP) and heart rate ranged from 126/61 to 164/93 and 72 to 98 bpm, respectively, during the entire monitoring period, and did not drop significantly during the neurological worsening. There were no clinical signs or lab results suggestive of a systemic inflammatory response syndrome (e.g., elevated white blood cell count or CRP, abnormal body temperature). An urgent CT (within 15 minutes after neurological worsening) ruled out intracerebral hemorrhage and edema progression. TCD monitoring showed persisting right MCA occlusion (TIBI score 2) and paradoxical as well as transient velocity decreases (>10 cm/s) during hypoventilation episodes consistent with intracranial blood flow steal. Because of continuation of excessive sleepiness with apnea periods, the patient was placed on biphasic positive airway pressure (BiPAP). Within the next hour, the patient improved to an NIHSS score of 13, and TCD showed continuing improvement in flow velocities suggestive of slow and partial recanalization (Figure 1). Next day, CT demonstrated an infarction involving 1/3 of the MCA territory (Figure 1). On TCD, right MCA appeared completely recanalized (TIBI score 5, Figure 1). Further clinical workup revealed a moderate stenosis (50 to 69%) in the right internal carotid artery strongly suggesting large-artery thrombosis and artery-to-artery embolism as the likely mechanism of her stroke. 

BiPAP was transitioned to night-time ventilation only, and her neurological status improved to an NIHSS of 6 at discharge. One month later, she had a residual minor left-sided hemiparesis and was functionally independent (NIHSS 3, modified Rankin Scale 1). An overnight sleep study in our sleep-wake disorder center four months later demonstrated a significant OSA (apnea-hypopnea index > 10/h), which improved on continuous positive airway pressure.

## 3. Discussion

In this paper, an early neurological deterioration was noticed that was not related to proximal vessel patency changes but coincidentally with the development of drowsiness and sleep apnea. Noninvasive ventilatory correction was applied in the hyperacute phase of ischemic stroke, and this could have improved cerebral hemodynamics distal to the occlusion. The latter is likely to have occurred as evident from her fast neurological improvement on BiPAP without complete vessel recanalization. Although tPA infusion was bridged with an experimental protocol that involves a direct thrombin inhibitor, the specific agent here was not at question. Our case highlights possible significance of ventilatory management in acute stroke, and the magnitude at which ventilatory compromise can affect both routine treatment and any hyperacute clinical trial. 

OSA is frequently present in patients with acute ischemic stroke and associated with a higher likelihood of early clinical deterioration [1, 2]. However, the underlying pathophysiologic mechanism for irregular breathing and early neurological deterioration in acute ischemic stroke patients is poorly understood. Under ischemic conditions, cerebral vasomotor reserve may become exhausted with occurrence of an intracranial blood flow steal, and affected vessels are less responsive to vasodilator stimuli like carbon dioxide leading to an intracranial blood flow steal from the affected to the nonaffected side [6–8]. This intracranial hemodynamic steal phenomenon has been documented on continuous TCD monitoring in up to 14% of stroke patients, leading to clinical deterioration in 7% of cases (termed reversed Robin Hood syndrome) [7]. Moreover, patients with proximal arterial occlusion and excessive daytime sleepiness were more vulnerable to intracranial blood flow steal than patients without these conditions. 

We cannot rule out that early neurological deterioration in our case simply reflected a natural course of proximal MCA occlusion and failure of collateral flow [9]. However, at the time of neurological worsening, our patient developed excessive sleepiness with recurrent and witnessed episodes of hypoventilation/apnea. Unfortunately, we did not measure arterial blood gases as arterial stick after intravenous tPA infusion was deemed risky. Nevertheless, early clinical recovery after application of BiPAP may represent a clinical surrogate of arterial carbon dioxide correction and increased blood flow in vessels supplying ischemic tissue. We considered that early clinical recovery might also have reflected a partial recanalization after intravenous thrombolysis. However, at the time of significant clinical improvement, MCA was still occluded. 

In conclusion, noninvasive ventilatory correction should be considered more aggressively as a complementary treatment option in selected acute stroke patients. Further research is needed to determine whether early initiation of this treatment may improve functional outcomes and unmask the true potential of other therapies.
